# Depletion of Alveolar Macrophages Does Not Prevent Hantavirus Disease Pathogenesis in Golden Syrian Hamsters

**DOI:** 10.1128/JVI.00304-16

**Published:** 2016-06-24

**Authors:** Christopher D. Hammerbeck, Rebecca L. Brocato, Todd M. Bell, Christopher W. Schellhase, Steven R. Mraz, Laurie A. Queen, Jay W. Hooper

**Affiliations:** a Virology Division, United States Army Medical Research Institute of Infectious Diseases (USAMRIID), Ft. Detrick, Maryland, USA; b Pathology Division, United States Army Medical Research Institute of Infectious Diseases (USAMRIID), Ft. Detrick, Maryland, USA; c Veterinary Medicine Division, United States Army Medical Research Institute of Infectious Diseases (USAMRIID), Ft. Detrick, Maryland, USA; University of Iowa

## Abstract

Andes virus (ANDV) is associated with a lethal vascular leak syndrome in humans termed hantavirus pulmonary syndrome (HPS). The mechanism for the massive vascular leakage associated with HPS is poorly understood; however, dysregulation of components of the immune response is often suggested as a possible cause. Alveolar macrophages are found in the alveoli of the lung and represent the first line of defense to many airborne pathogens. To determine whether alveolar macrophages play a role in HPS pathogenesis, alveolar macrophages were depleted in an adult rodent model of HPS that closely resembles human HPS. Syrian hamsters were treated, intratracheally, with clodronate-encapsulated liposomes or control liposomes and were then challenged with ANDV. Treatment with clodronate-encapsulated liposomes resulted in significant reduction in alveolar macrophages, but depletion did not prevent pathogenesis or prolong disease. Depletion also did not significantly reduce the amount of virus in the lung of ANDV-infected hamsters but altered neutrophil recruitment, MIP-1α and MIP-2 chemokine expression, and vascular endothelial growth factor (VEGF) levels in hamster bronchoalveolar lavage (BAL) fluid early after intranasal challenge. These data demonstrate that alveolar macrophages may play a limited protective role early after exposure to aerosolized ANDV but do not directly contribute to hantavirus disease pathogenesis in the hamster model of HPS.

**IMPORTANCE** Hantaviruses continue to cause disease worldwide for which there are no FDA-licensed vaccines, effective postexposure prophylactics, or therapeutics. Much of this can be attributed to a poor understanding of the mechanism of hantavirus disease pathogenesis. Hantavirus disease has long been considered an immune-mediated disease; however, by directly manipulating the Syrian hamster model, we continue to eliminate individual immune cell types. As the most numerous immune cells present in the respiratory tract, alveolar macrophages are poised to defend against hantavirus infection, but those antiviral responses may also contribute to hantavirus disease. Here, we demonstrate that, like in our prior T and B cell studies, alveolar macrophages neither prevent hantavirus infection nor cause hantavirus disease. While these studies reflect pathogenesis in the hamster model, they should help us rule out specific cell types and prompt us to consider other potential mechanisms of disease in an effort to improve the outcome of human HPS.

## INTRODUCTION

Hantaviruses are enveloped members of the family Bunyaviridae that contain a trisegmented, negative-sense, single-strand RNA genome. The three gene segments, L, S, and M, encode the RNA polymerase, nucleoprotein (NP), and envelope glycoproteins (G1 and G2), respectively. While these pathogens are carried chronically and asymptomatically in rodent hosts, in humans hantaviruses cause two unique vascular-leak syndromes that cover a spectrum of severity ranging from proteinuria to pulmonary edema and frank hemorrhage (1–4). Old World hantaviruses, including Puumala virus (PUUV), Dobrava virus (DOBV), Seoul virus (SEOV), and Hantaan virus (HTNV), have been associated with a mild-to-severe disease known as hemorrhagic fever with renal syndrome (HFRS). HFRS has a case-fatality rate between <0.1% and 15% and is characterized by fever, vascular leakage resulting in hemorrhagic manifestations, and renal failure. New World hantaviruses have been associated with a highly lethal disease, hantavirus pulmonary syndrome (HPS). HPS caused by the most prevalent North American and South American hantaviruses, Sin Nombre virus (SNV) and Andes virus (ANDV), respectively, has a case-fatality rate of 30 to 50% and is characterized by fever and vascular leakage resulting in noncardiogenic pulmonary edema followed by shock. Hantaviruses alter the barrier properties of the microvascular endothelial cells that they infect, causing vascular leakage in the kidneys or lungs (5). The specific mechanism underlying this endothelium dysfunction remains unknown, but hantavirus infection of endothelial cells is nonlytic, suggesting that other factors, possibly host derived, render the endothelium unable to regulate barrier integrity, leading to pulmonary edema (6).

While hantaviruses are known to cause disease by multiple routes of infection (5), the predominant route of human exposure is thought to be inhalation of excreta from infected rodent hosts (reviewed in references 6 and 7), suggesting that cells in the alveoli may play an important role in clearing, or alternatively, contributing to disease caused by aerosolized hantaviruses. Alveolar macrophages (AMθ) are found in the alveoli and alveolar ducts of the lung and represent the first line of defense against many airborne pathogens (8). Not only are they crucial regulators of immune system activity through their secretion of either pro- or anti-inflammatory cytokines, but also they are vitally important in the maintenance and remodeling of lung tissue via the production of growth factors, cytokines, and proteinases and can play a key role in the generation of protective cellular immune responses following intranasal vaccination (9–11). Activated AMθ are known to provide a critical element of protection against pathogens (12, 13) by releasing chemokines that recruit other innate immune cell types to areas of infection and secreting antiviral cytokines. However, activation of AMθ can also contribute to pathology by releasing the same cytokines that are important in providing protection from pathogens (14–17). Alveolar macrophages secrete multiple cytokines when activated, including interleukin-1 (IL-1), IL-6, IL-8, transforming growth factor β (TGF-β), inducible nitric oxide synthase (iNOS), and tumor necrosis factor alpha (TNF-α). Notably, the production of TNF-α further upregulates the release of other proinflammatory cytokines such as IL-1β, IL-6, and IL-8, which contribute to the initiation of adaptive immune responses (18). While these cytokines and chemokines act locally to choreograph immune responses that are important for protection against pulmonary pathogens, a number of these cytokines have been shown to promote vascular permeability and pulmonary edema, which are the hallmarks of pathogenic hantavirus infection (19–23). Correspondingly, studies of humans infected with hantavirus have detected high titers of proinflammatory and vasoactive cytokines in lung tissue of hantavirus pulmonary syndrome patients and high numbers of cytokine-producing cells correlated with the severity of HPS pathology (24). Moreover, systemic levels of inflammatory cytokines have also been reported in plasma of patients with hemorrhagic fever with renal symptoms (25), suggesting a role for these cytokines in disease pathogenesis.

Alveolar macrophages are known to be permissive to hantavirus infection (26, 27) but do not appear to be primary targets of infection, as hantavirus replication in alveolar macrophages is less efficient than in endothelial cells. Furthermore, AMθ have been found to be associated with hantavirus antigen in cases of human HPS (28) caused by SNV or in cases of “European HPS” following PUUV infection (29), but it isn't clear if that is a result of direct infection of alveolar macrophages or a result of phagocytosis. Despite these associations, hantavirus infection of human AMθ induced only modest antiviral responses and cell culture supernatants from SNV-infected AMθ failed to cause increased permeability of endothelial cell monolayers (27), suggesting that soluble mediators secreted by infected AMθ do not contribute to hantavirus disease.

ANDV causes a lethal disease in adult Syrian hamsters (30) that resembles HPS in humans, including the clinical signs of dyspnea, fluid in the pleural cavity, histopathology in the lungs and spleen, the disease incubation period, and the rapid progression from first signs to death (31). To determine if AMθ contribute to hantavirus disease in hamsters, we depleted AMθ using clodronate-encapsulated liposomes, delivered prior to ANDV challenge. Clodronate treatment significantly reduced the percentage and number of AMθ in hamster bronchial alveolar lavage (BAL) fluid during intramuscular (i.m.) and intranasal (i.n.) ANDV challenge but had little effect on disease pathogenesis. Depletion did result in a slightly more rapid and uniform disease course during intranasal infection, suggesting that AMθ may provide some protection against exposure to airborne ANDV, but overall, these data suggest that AMθ do not directly contribute to hantavirus disease pathogenesis in the Syrian hamster model of human hantavirus pulmonary syndrome.

## MATERIALS AND METHODS

### Virus, cells, and medium.

ANDV strain Chile-9717869 (30) was propagated in Vero E6 cells (Vero C1008, ATCC CRL 1586). The preparation of twice-plaque-purified ANDV stock has been described previously (30). Cells were maintained in Eagle's minimum essential medium with Earle's salts containing 10% fetal bovine serum (FBS), 10 mM HEPES (pH 7.4), 1× penicillin-streptomycin (Invitrogen), and gentamicin sulfate (50 μg/ml) at 37°C in a 5% CO_2_ incubator.

### Challenge with hantavirus.

Female Syrian hamsters 6 to 8 weeks of age (Harlan, Indianapolis, IN) were anesthetized by inhalation of vaporized isoflurane using an IMPAC 6 veterinary anesthesia machine. For i.m. challenges, anesthetized hamsters were injected with 80 PFU (10 50% lethal doses [LD_50_]) of virus diluted in phosphate-buffered saline (PBS; 0.2 ml, caudal thigh) delivered with a 1-ml syringe with a 25-gauge, five-eighths-inch needle. For i.n. challenges, anesthetized hamsters were administered 50 μl delivered as 25 μl per naris with a plastic pipette tip (total amount of ANDV, 4,000 PFU; 42 LD_50_). Groups of 8 hamsters were typically used for experimental treatments, unless otherwise stated. All work involving hamsters was performed in an animal biosafety level 4 (ABSL-4) laboratory. Hamsters were observed two to three times daily. Euthanasia was performed on animals meeting early endpoint criteria.

### Macrophage depletion.

Clodronate-encapsulated liposomes (5 mg/ml clodronate; Clodrosome) (referred to here as liposomal clodronate) and control PBS-encapsulated liposomes (Encapsome) (referred to here as control liposome) were purchased from Encapsula Nano Sciences. Hamsters were anesthetized using 0.2 ml/100 g of body weight rat KAX (ketamine-acepromazin-xylazine) administered by i.m. injection. Each animal was then placed in a dorsal recumbent position, and an otoscope (Welch Allyn) was used to visualize the vocal folds. The vocal folds were numbed by topically administering a 2% lidocaine HCl jelly (Akorn), and then a 16-gauge 1.25-in Surflo catheter (Terumo) was passed between the vocal folds. Hamsters were then treated with either 0.2 ml liposomal clodronate or 0.2 ml control liposome by attaching a loaded syringe to the catheter and aspirating the contents into the lung.

### Flow cytometry analysis.

Hamsters were deeply anesthetized (0.4 ml Rat KAX/100 g) and then extensively perfused with sterile saline (Baxter) before being euthanized. To isolate alveolar macrophages, animals were placed in a dorsal recumbent position, and then a midline neck incision was made and downward dissection was performed carefully so that the trachea was exposed. A second incision was made near the xyphoid process, and scissors were used to remove the rib cage and expose the lungs. Care was taken to ensure the lungs were not damaged. A 16-gauge 1.25-in catheter was inserted into the trachea, and the lungs were lavaged 3 times using 1 ml of a 0.02% EDTA solution. BAL samples were then centrifuged at 514 × *g* for 5 min. Cells were then collected and washed twice in PBS containing 2% FBS. In some experiments, cells were incubated at 4°C for 15 min in a blocking buffer consisting of PBS containing 2% FBS and 2% normal rat serum (Sigma-Aldrich) prior to staining with antibody. Approximately 10^6^ cells were stained with mouse-anti-hamster MARCO (32) (clone PAL-1; 10 μg/100 μl; AbD Serotec) followed by anti-mouse IgM (clone RMM-1; 0.4 μg/ml; BioLegend) for 15 to 20 min at 4°C. Stained cells were then fixed in Cytofix buffer (BD Biosciences) for 15 min at 4°C before being analyzed on a FACSCalibur flow cytometer (BD Biosciences) using CellQuest software (BD Biosciences) or FACSCanto II flow cytometer (BD Biosciences) using FACsDiva software (BD Biosciences). AM and neutrophil cell numbers in BAL fluid preparations were mathematically determined by comparing cell numbers to numbers of PKH26 reference microbeads (Sigma) using the following formula: *N*_cells/ml_ = (*N*_cell events_ × dilution factor/*N*_bead events_ × dilution factor) × *N*_beads/ml_, where *N*_cells/ml_ is the number of cells per milliliter, *N*_cell events_ is the number of cell events, *N*_beads/ml_ is the number of beads per milliliter, and *N*_bead events_ is the number of bead events. Data were analyzed using FlowJo software (Treestar).

### Plaque assay.

Hantavirus plaque assays were performed as previously described (33).

### Isolation of RNA and real-time PCR.

Approximately 250 mg of lung tissue was homogenized in 1.0 ml TRIzol reagent using gentleMACS M tubes and a gentleMACS dissociator on the RNA setting. RNA was extracted from TRIzol samples as recommended by the manufacturer. The concentration of the extracted RNA was determined using a NanoDrop 8000 instrument and raised to a final concentration of 10 ng/μl. Real-time PCR was conducted on a Bio-Rad CFX thermal cycler using an Invitrogen Power SYBR green RNA-to-*C_T_* one-step kit according to the manufacturer's protocols. Primer sequences are as follows (26): ANDV S 41F, 5′-GAA TGA GCA CCC TCC AAG AAT TG-3′; ANDV S 107R, 5′-CGA GCA GTC ACG AGC TGT TG-3′. Cycling conditions were 30 min at 48°C, 10 min at 95°C, and 40 cycles of 15 s at 95°C and 1 min at 60°C. Data acquisition occurred following the annealing step.

### Hamster cytokine ELISAs.

Anti-hamster MIP-1α (MBS033532), MIP-2 (MBS006761), TNF-α (MBS046042), and vascular endothelial growth factor A (VEGF-A) (MBS024541) enzyme-linked immunosorbent assay (ELISA) kits were purchased from MyBioSource and were used according to the manufacturer's recommendations.

### Preparation of tissues for histology.

Tissues were fixed in 10% neutral buffered formalin, trimmed, processed, embedded in paraffin, cut at 5 to 6 μm, and stained with hematoxylin and eosin (H&E) for histopathology analysis. To determine the presence of ANDV antigens in association with alveolar macrophages or colocalized with endothelial cells, serial sections were then stained as follows. For ANDV immunohistochemistry, a monoclonal antibody (USAMRIID number 1244) against ANDES virus was used on all tissue slides. Normal mouse IgG was used as the negative serum control for the control slides. Briefly, the unstained sections were deparaffinized, rehydrated, and pretreated with Tris-EDTA buffer for 30 min at 95 to 100°C. Slides were rinsed, and a serum-free protein block with 5% horse serum was applied for 30 min. The monoclonal antibody was then applied to the tissue at a dilution of 1:1,200 and incubated for 1 h at room temperature. The slides were then treated with alkaline phosphatase-labeled secondary mouse IgG antibody (catalog number MP-5402; Vector Laboratories, Burlingame, CA) for 30 min at room temperature. All slides were exposed to ImmPACT Vector Red (catalog number SK-5105; Vector Laboratories, Burlingame, CA) substrate-chromogen for 30 min, rinsed, counterstained with hematoxylin, dehydrated, and coverslipped with Permount (catalog number SP15-500; Fisher). For CD31 immunohistochemistry, an immunoperoxidase assay was performed using a rabbit anti-CD31 polyclonal antibody (catalog number ab28364; Abcam). A normal rabbit IgG was used as the negative serum control for the control slides. Briefly, the unstained sections were deparaffinized, rehydrated, subjected to a methanol hydrogen peroxide block, rinsed, and pretreated with Tris-EDTA buffer for 30 min at 95 to 100°C. Slides were rinsed, and a serum-free protein block with 5% goat serum was applied for 30 min. The polyclonal antibody was then applied to the tissue at a dilution of 1:75 and incubated overnight at room temperature. The slides were then treated with the EnVision horseradish peroxidase-labeled secondary antibody (catalog number K4007; Dako, Carpinteria, CA) for 30 min at room temperature. All sections were exposed to a DAB (3,3-diaminobenzidine) substrate-chromogen for 5 min, rinsed, counterstained with hematoxylin, dehydrated, and coverslipped with Permount.

### Statistical analysis.

Survival curves were compared with Kaplan-Meier survival analysis with log rank comparisons and Dunnett's correction. Comparisons of viral genome, infectious virus, alveolar macrophage, and neutrophil percentages and numbers and ELISA cytokine titers were done using a one-way analysis of variance (ANOVA) with Tukey's multiple-comparison test. *P* values of less than 0.05 were considered significant. Analyses were conducted using GraphPad Prism (version 5).

### Ethics statement.

Research at the U.S. Army Medical Research Institute of Infectious Diseases (USAMRIID) was conducted under an Institutional Animal Care and Use Committee (IACUC)-approved protocol in compliance with the Animal Welfare Act, PHS Policy, and other federal statutes and regulations relating to animals and experiments involving animals. The facility where this research was conducted is accredited by the Association for Assessment and Accreditation of Laboratory Animal Care, International, and adheres to the principles stated in the Guide for the Care and Use of Laboratory Animals, National Research Council, 2011.

## RESULTS

### Depletion of AMθ does not prevent disease following intramuscular ANDV challenge.

**FIG 1:**
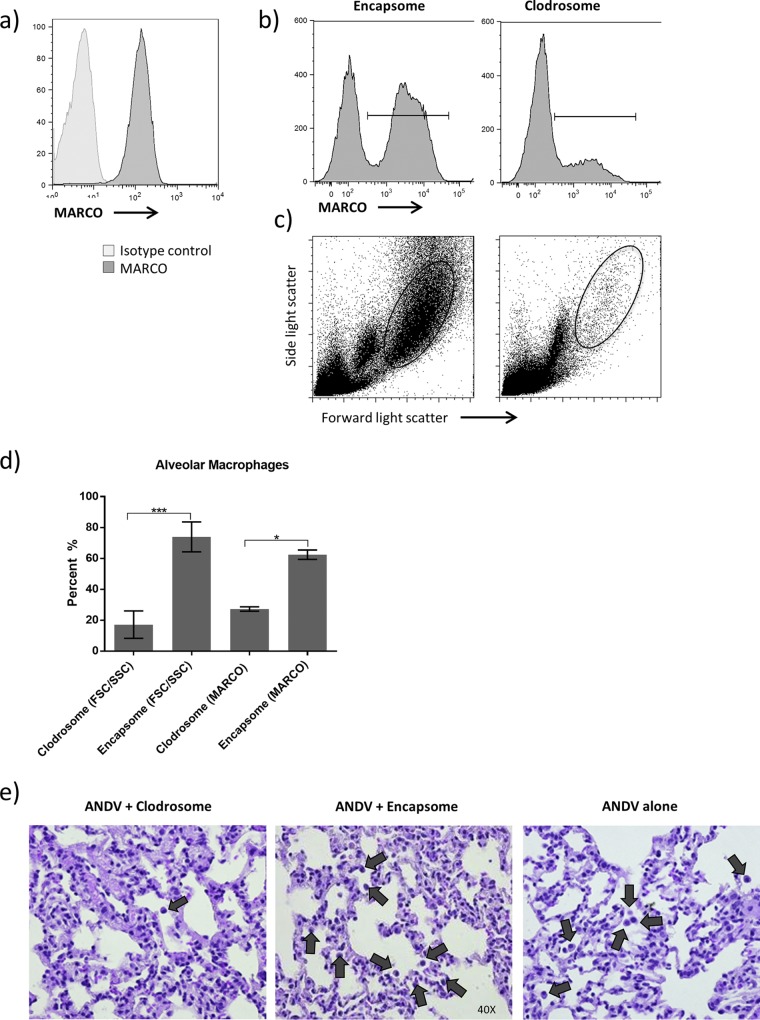
Identification and depletion of AMθ in Syrian hamsters. (a) AMθ from Syrian hamster BAL fluid were analyzed for the expression of the MARCO scavenger receptor by flow cytometry. Hamsters were treated intratracheally with liposomal clodronates or control liposomes on days −3 and −1 prior to an 80-PFU ANDV i.m. challenge. Ten days post-ANDV challenge, the ability of Encapsome or liposomal clodronate treatment to deplete AMθ was determined by analyzing the percentage of MARCO^+^ cells (b) or FSC^hi^/SSC^hi^ cells (c) in hamster BAL samples. The percentage of MARCO^+^ cells or FSC^hi^/SSC^hi^ cells was then quantified (d) (*, *P* < 0.05; ***, *P* < 0.001). (e) Staining of lungs of day 10 ANDV-infected hamsters with H&E (total magnification, ×400). Sections (5 to 6 μm) of one of the cranial lung lobes was stained with H&E to visually determine if the number of alveolar macrophages is affected when treated with liposomal clodronate or control liposome or when left untreated. Black arrows indicate alveolar macrophages.

**FIG 2:**
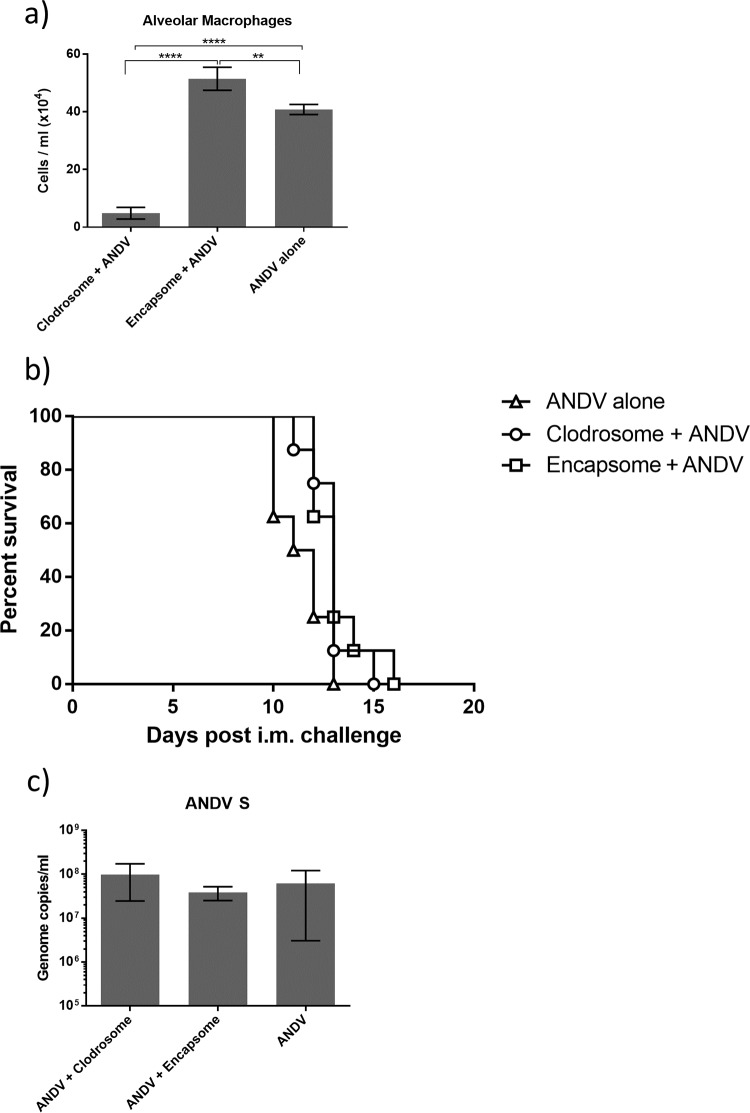
Depletion of AMθ does not prevent disease following intramuscular ANDV challenge. Hamsters were treated intratracheally with liposomal clodronate or control liposome on days −3 and −1 or were left untreated. On day 0, all hamsters were challenged with 80 PFU ANDV by intramuscular infection. (a) Ten days post-ANDV challenge, the number of AMθ was determined by flow cytometry by gating on FSC^hi^/SSC^hi^ cells (as described for Fig. 1) in hamster BAL samples (**, *P* < 0.01; ****, *P* < 0.0001). (b) The depletion of AMθ did not prevent disease in hamsters. Lung tissue specimens isolated from all hamsters 10 days postchallenge were evaluated for viral genome (c) by real-time (RT)-PCR (not significant).

Liposomal clodronate has been used extensively to deplete AMθ in many animal models, including the Syrian hamster (34). In the Syrian hamster, AMθ were identified as high forward light scatter, high side light scatter (FSC^hi^SSC^hi^), MARCO-expressing cells in hamster BAL fluid (Fig. 1a) and intratracheal administration of clodronate-encapsulated liposomes was found to effectively reduce the number of AMθ in hamsters during ANDV infection as determined by a reduction in either MARCO^+^ cells (Fig. 1b and d) or FSC^hi^SSC^hi^ cells (Fig. 1c and d) and histolopathologic analysis of hamster lung tissue (Fig. 1e) during ANDV infection of hamsters. To begin to understand the role that AMθ play during ANDV disease pathogenesis, hamsters were treated intratracheally with clodronate-encapsulated liposomes (Clodrosomes) or control PBS-encapsulated liposomes (Encapsomes) on days −3 and −1. One group of hamsters was left untreated. Hamsters were then challenged with 80 PFU (10 LD_50_) ANDV i.m. Ten days later, the number and percentage of AMθ in hamster BAL fluid were determined. Control liposome treatment did induce an increase in the number of alveolar macrophages (Fig. 2a), but by comparison, clodronate treatment resulted in a significant reduction in the total number of alveolar macrophages (Fig. 2a). Macrophage depletion did not prevent disease in hamsters (Fig. 2b) or significantly alter the mean time to death (liposomal clodronate, 12.88 days; control liposome, 13.13 days; untreated, 11.38 days). The mean time to death following control liposome treatment was significantly longer than that in untreated animals (13.13 days versus 11.38 days; *P* = 0.05) but not longer than that in clodronate-treated animals. Depletion of AMθ also did not result in increased ANDV titers in the lung as measured by PCR (Fig. 2c). These data suggest that despite becoming activated, AMθ are not important for protection against an intramuscular ANDV challenge, nor do they contribute to disease pathogenesis following intramuscular challenge.

**FIG 3:**
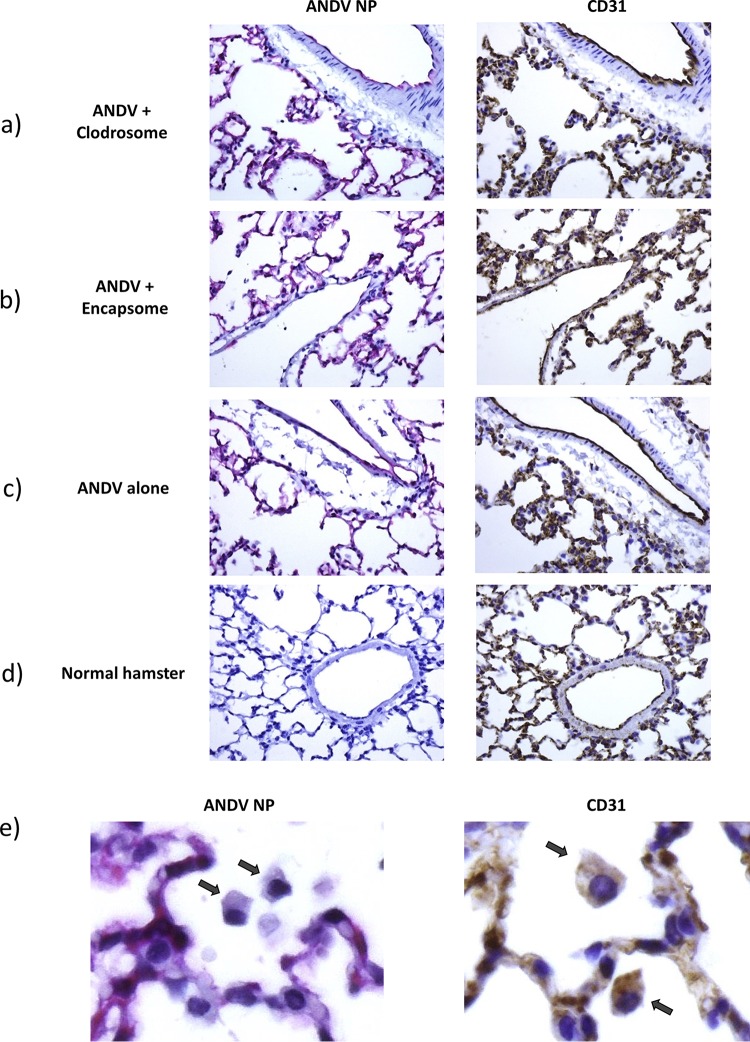
Depletion of AMθ does not alter the localization of ANDV NP to CD31 positive endothelial cells. Serial sections (5 to 6 μm) from one of the cranial lobes of day 10 i.m. ANDV-infected hamsters treated with liposomal clodronate (a) or control liposome (b), untreated ANDV-infected hamsters (c), or normal uninfected hamsters (d) were stained with antibodies specific for CD31 (DAB; brown) or ANDV NP (alkaline phosphatase; red). ANDV NP staining colocalized to CD31-positive cells in adjacent serial sections. Normal hamster tissue remained negative for ANDV NP. No differences were seen in the pattern of CD31 and/or ANDV NP staining across treatment groups. (e) Alveolar macrophages from untreated ANDV-infected hamsters were evaluated for the presence of ANDV NP and CD31. All were found to be CD31 positive, but no evidence of positive staining for ANDV NP was detected.

Serial sections of lung tissue from these groups further revealed the presence of ANDV antigen colocalized to CD31-positive endothelial cells in both capillaries and larger vessels (Fig. 3a to d). Regardless of treatment, no differences were observed in the pathogenesis of HPS-like disease in ANDV-infected hamsters. Hamsters in all groups exhibited signs of mild to moderate inflammation, interstitial pneumonia, alveolar fibrin deposition, and edema characteristic of ANDV infection.

Also noticed were multifocal foci of neutrophilic inflammation, along with mesothelial hypertrophy and atelectasis. The presence of necrotic/apoptotic debris was rare. Consistent with the detection of viral genome by PCR, immunohistochemistry analysis revealed little to no difference in overall viral load within endothelial cells following clodronate treatment. Interestingly, all identifiable alveolar macrophages found in ANDV-infected hamsters were negative for Andes virus although positive for cytoplasmic CD31 staining (Fig. 3e).

### Depletion of AMθ does not prevent disease following intranasal ANDV challenge.

**FIG 4:**
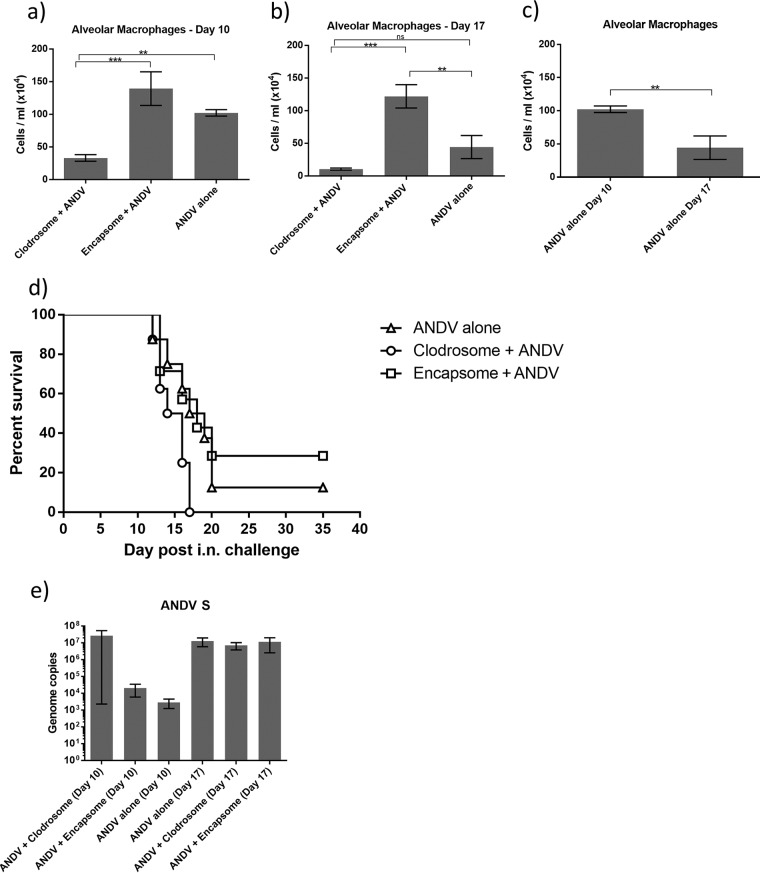
Depletion of alveolar macrophages does not prevent disease following intranasal ANDV challenge. Hamsters were treated intratracheally with liposomal clodronate or control liposome on days −3 and −1 or were left untreated. On day 0, all hamsters were challenged with 4,000 PFU ANDV by intranasal infection. (a and b) Ten days (a) and 17 days (b) post-ANDV challenge, the number of AMθ was determined by flow cytometry by gating on FSC^hi^/SSC^hi^ cells (as described for Fig. 1) in hamster BAL samples (**, *P* < 0.01; ***, *P* < 0.001; ns, not significant). (c) The numbers of AMθ on days 10 and 17 in untreated, ANDV-challenged hamsters were directly compared (**, *P* < 0.01). (d) The depletion of AMθ did not prevent disease in hamsters. All surviving animals seroconverted, indicating that they had been exposed to virus (data not shown). (e) Lung tissue isolated from all hamsters 10 and 17 days postchallenge were evaluated for viral genome by RT-PCR.

Alveolar macrophages are more likely to be involved in the defense against airborne pathogens. To understand the protective or pathogenic responses that AMθ elicit to inhaled hantaviruses, AMθ were depleted prior to intranasal ANDV challenge. Groups of hamsters were treated intratracheally with liposomal clodronate or control liposome on days −3 and −1. One group of hamsters were left untreated. Hamsters were then challenged with 4,000 PFU (42 LD_50_) ANDV i.n. Ten and 17 days later, the number and percentage of AMθ in hamster BAL fluid were determined. Similar to what was seen after intramuscular challenge, 10 days after intranasal challenge the percentage and total number of AMθ in untreated or control liposome-treated animals were comparable (Fig. 4a). control liposome treatment resulted in a trend toward increased numbers of AMθ compared to untreated animals, but this difference was not significant. By comparison, liposomal clodronate treatment resulted in a significant reduction in both the percentage and total number of AMθ. The percentage of AMθ remained significantly reduced following liposomal clodronate treatment 17 days after intranasal challenge (Fig. 4b). Surprising, though, was the observation that the number of AMθ at day 17 in the untreated group was significantly lower than the number of AMθ in the untreated group at day 10 (Fig. 4c). This phenomenon was seen only in the untreated groups, as the number of AMθ in the control liposome-treated animals remained similar between days 10 and 17. Although the total number of AMθ in the liposomal clodronate-treated animals was lower on day 17 than on day 10, the difference between the number of AMθ in the liposomal clodronate-treated animals and untreated animals on day 17 was not significant. However, the difference in the number of AMθ in the control liposome-treated animals and untreated animals on day 17 was significant. The reduction in AMθ did not prevent disease in hamsters (Fig. 4d), but depletion did result in a more uniform and slightly more rapid disease course (mean time to death: liposomal clodronate, 14.75 days; control liposome, 21.43 days; no treatment, 19.13 days). ANDV titers in the lung were not significantly different in liposomal clodronate-treated hamsters at either day 10 or day 17 as determined by the presence of viral genome measured by PCR (Fig. 4e). However, there was a trend toward increased ANDV M copy number in the lungs of liposomal clodronate-treated animals 10 days postchallenge.

**FIG 5:**
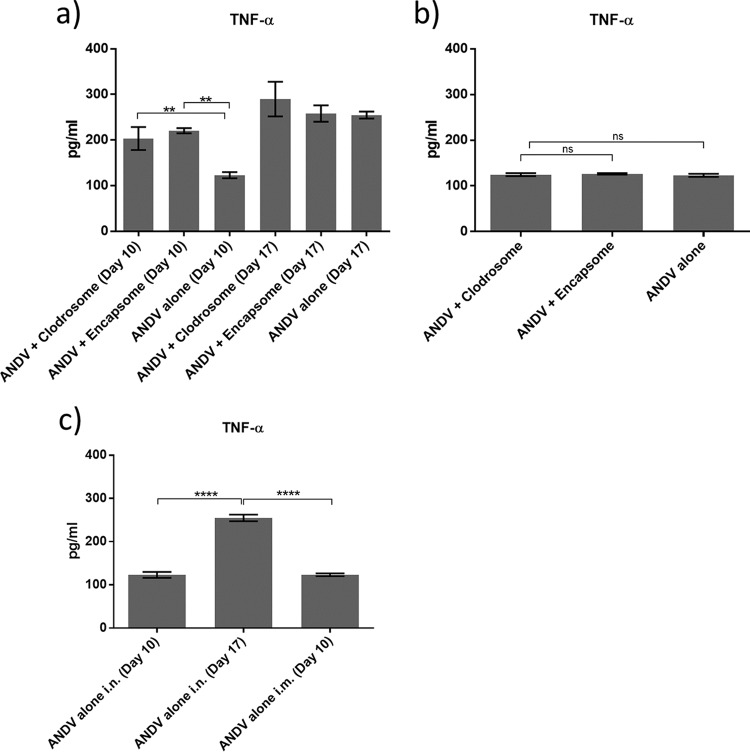
Depletion AMθ alters TNF-α expression but only early after intranasal ANDV challenge. Hamsters were treated intratracheally with liposomal clodronate or control liposome on days −3 and −1 or were left untreated. On day 0, all hamsters were challenged either with 4,000 PFU ANDV by intranasal infection or with 80 PFU ANDV by intramuscular infection. BAL samples were collected from all hamsters 10 and 17 days after intranasal challenge (a) or 10 days after intramuscular challenge (b), and TNF-α expression was analyzed by ELISA. The depletion of AMθ resulted in increased TNF-α levels 10 days after intranasal ANDV challenge (a) (**, *P* < 0.01) but did not affect TNF-α levels 17 days after intranasal challenge or 10 days after intramuscular ANDV challenge (b). (c) TNF-α expression levels in BAL samples from untreated ANDV-challenged hamsters following either intranasal or intramuscular virus challenge were directly compared (****, *P* < 0.0001).

**FIG 6:**
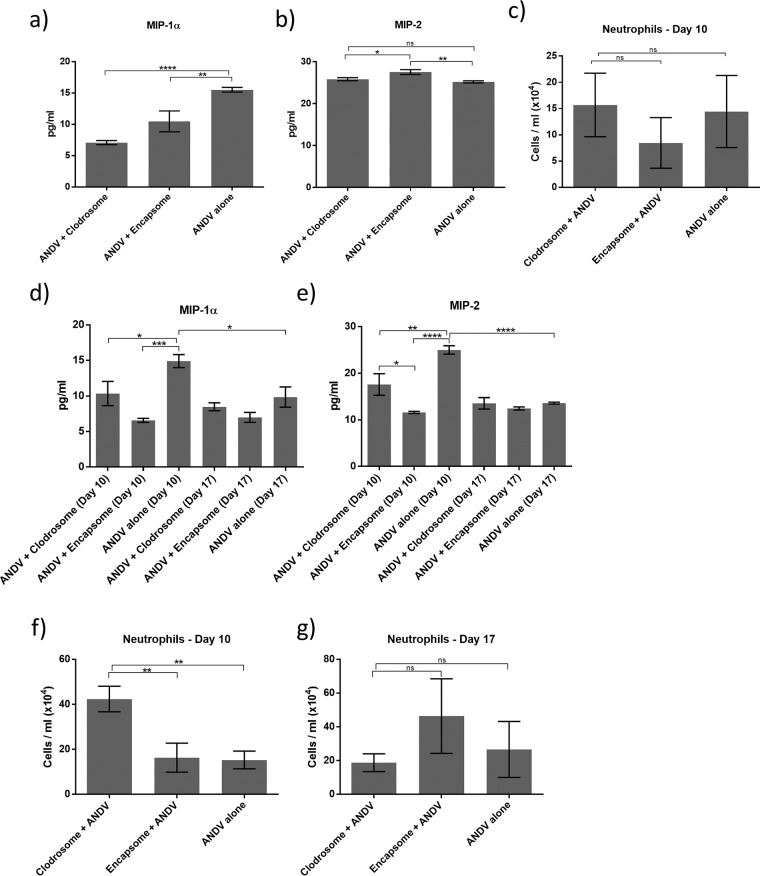
Depletion of AMθ alters neutrophil recruitment and neutrophil chemoattractant expression early after intranasal ANDV challenge. BAL samples were collected from all hamsters 10 days after intramuscular challenge (a to c) or 10 and 17 days after 4,000 PFU intranasal ANDV challenge (d to g) and were analyzed for the presence of neutrophils by flow cytometry and MIP-1α and MIP-2 by ELISA. Both liposomal clodronate and control liposome treatments resulted in a decrease in MIP-1α (a) but did not alter MIP-2 expression (b) or the number of neutrophils (c) compared to untreated hamsters after intramuscular challenge. liposomal clodronate and control liposome treatments resulted in a decrease in both MIP-1α (d) and MIP-2 expression (e). liposomal clodronate treatment resulted in an increase in the number of neutrophils in hamster BAL fluid on day 10 (f) but not on day 17 (g) after intranasal challenge (*, *P* < 0.05; **, *P* < 0.01; ***, *P* < 0.001; ****, *P* < 0.0001; ns, not significant).

TNF-α levels in hamster BAL samples were significantly higher at the peak of disease than at earlier time points (Fig. 5a; day 17 versus day 10), but AMθ depletion affected TNF-α expression only early after infection. Interestingly, treatment with either liposomal clodronate or control liposome resulted in an increase in detected TNF-α compared to that in untreated hamsters, which could reflect the higher numbers of neutrophils and AMθ present in the BAL samples from these groups, respectively (Fig. 4b and 6d). The reduction in AMθ did not reduce the amount of TNF-α detected in BAL samples 10 days after intramuscular challenge (Fig. 5b), which was similar to the amount of TNF-α detected in BAL samples 10 day after intranasal challenge. Remarkably, TNF-α levels at the peak of disease following intranasal challenge (day 17) were nearly twice those detected at the peak of disease following intramuscular challenge, possibly reflecting the differences in AMθ activation when virus is administered directly to the lung. These data suggest that AMθ do not contribute to disease pathogenesis but may contribute some degree of protection against intranasal ANDV challenge.

### Depletion of AMθ alters neutrophil recruitment early, but not late, after intranasal ANDV challenge.

Alveolar macrophages coordinate many aspects of immune responses to airborne pathogens, including the recruitment of other immune cell types such as neutrophils. At the peak of hantavirus disease following intramuscular infection with ANDV (day 10), we observed a decrease in the neutrophil chemoattractant MIP-1α in BAL samples from AMθ-depleted hamsters compared to untreated ANDV-infected hamsters, suggesting that AMθ may be an important source of MIP-1α during hantavirus infection in the lung (Fig. 6a). However, the expression of the neutrophil chemoattractant MIP-2 in BAL samples from all hamsters remained unchanged (Fig. 6b), and, correspondingly, there was also little significant change in the number of neutrophils found in hamster BAL samples 10 days after intramuscular challenge (Fig. 6c). By comparison, when we investigated the role of AMθ in the recruitment of neutrophils following intranasal challenge, we found that early in disease pathogenesis (day 10), MIP-1α and MIP-2 expression were reduced in both liposomal clodronate- and control liposome-treated hamsters compared to untreated ANDV-infected hamsters (Fig. 6d and e). We also found that control liposome treatment resulted in increased numbers of recruited AMθ (Fig. 4b) while liposomal clodronate treatment resulted in an increased number of neutrophils (Fig. 6f). At the peak of disease following intranasal ANDV challenge (day 17), the expression of MIP-1α and MIP-2 was reduced approximately 50% compared to the expression seen on day 10. Moreover, the expression of MIP-1α and MIP-2 was not dependent on treatment, as equivalent amounts of MIP-1α and MIP-2 were detected in BAL samples from all hamsters (Fig. 6d and e). Neutrophil numbers were reduced in liposomal clodronate-treated animals by day 17 compared to the numbers observed on day 10, but neutrophil numbers in the control liposome-treated and untreated hamsters remained virtually unchanged between days 10 and 17 (Fig. 6g). In contrast, AMθ numbers in control liposome-treated hamsters remained elevated on days 10 and 17 despite an overall drop in AMθ numbers in untreated hamsters (Fig. 4a and b). These data suggest that AMθ may regulate neutrophil recruitment to the lung early after hantavirus infection but do not contribute significantly to neutrophil recruitment toward the peak of disease pathogenesis.

### Depletion of AMθ alters VEGF-A expression early after intranasal ANDV challenge.

**FIG 7:**
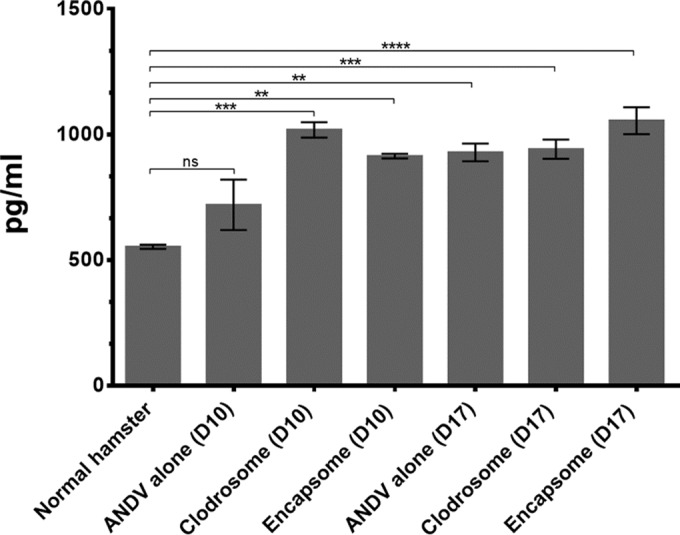
Depletion of AMθ alters VEGF-A expression early after intranasal ANDV challenge. BAL samples were collected from all hamsters 10 and 17 days after 4,000 PFU intranasal ANDV challenge and were analyzed for the presence of VEGF-A by ELISA. Both liposomal clodronate and control liposome treatments resulted in increased VEGF-A expression 10 days post-ANDV challenge comparable to VEGF-A levels found in all hamsters 17 days postchallenge (**, *P* < 0.01; ***, *P* < 0.001; ****, *P* < 0.0001; ns, not significant).

Recently, vascular endothelial growth factor (VEGF) has been hypothesized to play a role in hantavirus disease pathogenesis. Moreover, AMθ are known sources of VEGF. We therefore asked whether VEGF expression in the lungs of hamsters infected with ANDV was dependent on the presence of AMθ. Compared to normal uninfected hamsters, VEGF-A expression in the BAL of hamsters 10 days after intranasal ANDV challenge was only slightly, but not significantly, elevated (Fig. 7). Interestingly, at the time of peak disease on day 17, there was almost a 2-fold increase in VEGF-A protein. Macrophage depletion did not further enhance VEGF-A in BAL samples late into infection (day17), as we observed no difference in VEGF titers in ANDV-infected hamster BAL samples in the presence or absence of AMθ. However, when AMθ were depleted, VEGF-A expression in the BAL fluid on day 10 was equivalent to the amount of VEGF-A detected in all hamster BAL samples on day 17. A similar increase in VEGF was observed in control liposome-treated hamsters. These data demonstrate that VEGF expression is enhanced in hamsters infected with ANDV but suggest that while AMθ may regulate the expression of VEGF by other cell types in the lung, they are not a major source of VEGF during hantavirus disease pathogenesis.

## DISCUSSION

The human lung has a surface area of approximately 70 m^2^ and contains, on average, 480 million alveoli (35), which are in constant contact with the outside world. In this environment, homeostasis along the endothelial/epithelial border must be maintained to allow oxygen and carbon dioxide to freely exchange; however, these homeostatic mechanisms must remain pliable enough to allow immune responses to clear invading pathogens. Often considered the first line of defense against respiratory pathogens, the estimated 2 billion AMθ residing in the alveoli of the human lung (36) are uniquely juxtaposed to maintain lung homeostasis as well as orchestrate protection against airborne viruses and bacteria (37). In the case of hantaviruses, the predominant route of human exposure is thought to be inhalation of excreta from infected rodent hosts (reviewed in references 6 and 7), suggesting that alveolar macrophages may play an important role in clearing or, alternatively, contributing to disease caused by aerosolized hantaviruses. Albeit rare, several cases of hantavirus disease have been reported in humans following parenteral exposure resulting from the bite of infected rodents. In these cases, the disease resulting from parenteral infection is virtually identical to the disease following aerosol infection, the only differences being slight differences in disease kinetics. Similarly, Syrian hamsters experimentally infected with ANDV by intramuscular challenge develop a disease that is indistinguishable from the disease exhibited following intranasal exposure, the only differences being slight differences in disease kinetics and the number of PFU needed to develop uniformly lethal disease. Given the extensive similarities in disease, it is likely that the mechanism of disease or the cell type responsible for disease is identical regardless of the route of exposure. Consistent with this hypothesis, we demonstrate here that alveolar macrophages play only a marginal role in protecting hamsters from lethal hantavirus infection but do not contribute to the disease caused by either intranasal or intramuscular hantavirus infection.

Alveolar macrophages contribute to the defense against many aerosolized pathogens, and in many cases, these responses are critical for host protection. In models of vaccinia virus, respiratory syncytial virus (RSV), and influenza virus infection, the depletion of alveolar macrophages results in greater viral replication and dissemination and an overall increase in the severity of infection (9, 38–40). In some cases, the reduced levels of protection in the absence of alveolar macrophages are likely due to the impaired initiation of antiviral responses that result in abolished early cytokine and chemokine release and inhibited immune cell activation and recruitment (40, 41). In addition, Schneider et al. (42) demonstrated that when alveolar macrophages were depleted in mice prior to infection with influenza virus, the mice exhibited lower percent oxygen saturation (sO_2_) and oxygen partial pressure (pO_2_), arguing that AMθ are important for maintaining lung function during infection. However, the same mechanisms that alveolar macrophages use to protect against pathogens have also been implicated in causing disease and increased vascular permeability in models of acute lung injury caused by infectious disease agents, such as human metapneumovirus (hMPV) (43) and Pseudomonas aeruginosa (44), as well as chronic obstructive pulmonary disease (COPD) (45) and nonischemic inflammatory lung injury (46). Alveolar macrophages may also serve as a reservoir for pathogens such as hMPV (43), measles virus (47), and Legionella pneumophila (48) and may indirectly contribute to the pathogenesis of the different diseases caused by these pathogens by allowing their replication and dissemination. Still, the enhancement of disease in the presence of alveolar macrophages may not always reflect a direct contribution by AMθ by way of proinflammatory cytokines or angiogenic factors but may indirectly be the result of increased immune cell recruitment by AMθ as seen in mouse models of mouse hepatitis virus type 1 (MHV-1) infection (49).

The role that AMθ play in disease caused by classical hemorrhagic fever viruses is less well understood. Alveolar macrophages express the primary and secondary receptors (50) for both hantaviruses (αvβ3 integrin [51] and complement receptors 3 and 4 [CR3/CR4] [52]) and Ebola virus (DC-SIGN and DC-SIGNR [53]) and, correspondingly, are known to be permissive to hantavirus (26, 27) and Ebola virus infection (54). However, in these cases, infection is less efficient than, or fails to induce a sustained inflammatory response compared to, the primary targets of infection for these viruses, and there is no evidence that hantavirus infection of AMθ induces apoptosis. Antigens of yellow fever virus, which is transmitted by the bite of the Aedes aegypti mosquito, can be found inside the rough endoplasmic reticulum and Golgi complex of AMθ, suggesting that viral replication can occur in these cells (55, 56), but it is unknown whether AMθ play any role in disease pathogenesis other than acting as a virus reservoir. It is also unknown whether other hemorrhagic fever viruses that commonly target monocyte lineage cells, such as dengue virus and Crimean Congo hemorrhagic fever virus, target AMθ in a way that contributes to human disease. Alveolar macrophages are known to be permissive to hantavirus infection (26, 27), but it has been less clear to what extent AMθ act as a reservoir for hantaviruses. In some of the earliest characterizations of HPS, Mori and colleagues described finding viral antigen in only a low number of “large cells,” identified as macrophages, in the lung (24). Similarly, Li and Klein further demonstrated that while Norway rat alveolar macrophages could be infected with Seoul virus, their ability to support efficient replication of the virus was significantly reduced compared to endothelial cells (26).

Our analysis of ANDV nucleoprotein (NP) associated with AMθ was limited to intramuscular challenge, but a similar trend has been reported by Safronetz and colleagues, who noted that even during the end stages of hantavirus disease in hamsters infected intranasally with ANDV, only occasional alveolar luminal cells, most likely alveolar macrophages, were found to stain positive for hantavirus antigen (57). Coupled with this, we see no difference in HPS pathology in hamsters following either i.n. or i.m. challenge, and our analysis was done at the peak of disease pathogenesis following i.m. challenge (day 10), when ANDV NP staining of the lung endothelium was nearly continuous, suggesting that the likelihood of ANDV/AMθ interactions would be as high as that following intranasal challenge. Taken together, our observation that the depletion of AMθ resulted in no significant change in lung ANDV titers (Fig. 2c and 4e) and our inability to detect the presence of ANDV NP associated with hamster AMθ (Fig. 3e) suggest that AMθ do not serve as a primary reservoir for hantavirus replication and AMθ dysfunction due to direct hantavirus infection is unlikely.

How hamster AMθ respond to ANDV infection is not entirely clear. Alveolar macrophages, in general, need to walk a fine line between homeostasis and host defense to protect the host while preventing catastrophic inflammation. One way AMθ contribute to lung protection is by phagocytizing most of the particulate matter that enters the lungs. This suggests that they may also contribute to the clearance of ANDV from the lungs of hamsters. Early after intranasal ANDV challenge, we saw a trend toward increased detection of viral genome in hamsters that were depleted of AMθ, although depletion of AMθ does not significantly alter the amount of live virus or viral genome detected in lung tissue at late time points after infection (Fig. 3e and f). Hamsters devoid of AMθ also developed disease faster than untreated or control-treated ANDV-infected hamsters. Interestingly, the highest number of surviving animals was found in control liposome-treated animals (Fig. 3d). Correspondingly, this group also had greater numbers of AMθ than liposomal clodronate-treated or untreated animals (Fig. 3a to c). Like other models, this could suggest that early after infection, AMθ help prevent the spread of infection by reducing infectious virus in the lung and by so doing may help control the rate at which disease pathogenesis progresses. At later times after infection, similar levels of viral genome and/or infectious virus were found in the lung of all hamsters, supporting the argument that AMθ contribute more substantially to the immune response against ANDV early after infection but less so at later times once ANDV is primarily replicating in endothelial cells. This is also consistent with the reduced numbers of AMθ detected on day 17 compared to day 10.

A second way AMθ contribute to lung protection is by modulating immune responses in the lung (37, 58). In the presence of harmless particulates such as dust, they may go as far as suppressing antigen-specific adaptive immune responses either by directly suppressing tissue-resident T cells (59, 60) or by suppressing lung-resident dendritic cells (61), thus preventing them from migrating to draining lymph nodes and initiating immune responses. However, in the case of aerosolized pathogens, activation of AMθ results in a change in phenotype from a regulator of lung homeostasis to that of a cell capable of coordinating and participating in inflammatory immune responses (62, 63). Human AMθ make little TNF-α when exposed to SNV compared to lipopolysaccharide (LPS) (27), suggesting that hantavirus may be ineffective at activating AMθ. Correspondingly, we noticed little difference in the amount of TNF-α in the BAL fluid of hamsters in the presence or absence of AMθ following infection with ANDV intramuscularly (Fig. 4a) or at late times after intranasal infection (Fig. 4b). In the case of intramuscular injection of virus, it is not clear whether AMθ would be effectively stimulated since it is assumed that infection of the endothelium would occur directly via the blood and not by inhalation. We did observe an increase in the amount of TNF-α in BAL samples between day 10 and day 17 postintranasal challenge, indicating that an inflammatory response was occurring. However, at late times, depletion of macrophages did not reduce the amount of TNF-α detected, suggesting that AMθ are not a major source of TNF-α that late in infection. Somewhat surprisingly, at early times (day 10) after intranasal challenge, the depletion of AMθ resulted in an increase in TNF-α in BAL samples. One explanation is that in hamsters, AMθ are more prone to an immunosuppressive phenotype and by depleting them, other cell types, including neutrophils, endothelial cells, T cells, and epithelial cells are no longer prevented from producing TNF-α. The fact that control liposome-treated animals also had increased levels of TNF-α may be reflected in the increased numbers of AMθ induced by control liposome treatment. Resident AMθ at the time of control liposome treatment may retain their immunosuppressive phenotype, but any newly recruited AMθ could be expected to have a markedly different phenotype prior to adopting suppressive functions (64).

As immune sentinels, one of the primary roles of AMθ is to orchestrate immune responses by recruiting other immune cell types to the lung. In some models of acute lung injury (46, 65, 66), including pneumonia induced by LPS or P. aeruginosa pneumonia infection, depletion of AMθ attenuates the recruitment of neutrophils to the lung, indicating that AMθ can be a key source of neutrophil chemoattractants. Conversely, in other models, AMθ appear to play a greater role in the negative regulation of neutrophil migration in that the depletion of AMθ amplifies neutrophil recruitment (38, 44, 67–71). Neutrophils migrate in response to a number of chemoattractants (72) including CXCL2 (MIP-2 [mouse]/GROβ [human]) and CCL3 (MIP-1α), of which AMθ can be a major source (40, 73–75). Liposomal clodronate treatment resulted in a decrease in MIP-1α detected in BAL samples on day 10 following intramuscular ANDV challenge (Fig. 5a) and also resulted in a decrease in MIP-1α and MIP-2 on day 10 following intranasal challenge (Fig. 5b). Surprisingly, the decrease in MIP-1α and MIP-2 in liposomal clodronate-treated animals following intranasal challenge was accompanied by an increase in BAL neutrophils (Fig. 5d). Moreover, control liposome treatment also resulted in decreases in MIP-1α and MIP-2 but recruitment of new AMθ rather than neutrophils. One possible explanation for this apparent paradox is that AMθ are an important source of MIP-1α and MIP-2 in the hamster but hamster neutrophils preferentially respond to other chemokines, such as monocyte chemoattractant protein-1 (MCP-1) or KC, that may be more abundant in the absence of MIP-1α and MIP-2. Alternatively, MIP-1α and MIP-2 are preferentially secreted by cell types other than neutrophils in the hamster and the presence of AMθ suppresses neutrophil migration. When alveolar macrophages are depleted, neutrophils freely migrate to the lung and act as a MIP-1α/MIP-2 “sponge” that soaks up free chemokine and reduces the overall levels of bioavailable MIP-1α/MIP-2. A similar explanation could hold true for the decreased abundance of MIP-1α and MIP-2 following control liposome treatment in which newly arrived AMθ act as the chemokine sponge. A closer analysis of the kinetics of MIP-1α and MIP-2 expression following alveolar macrophage depletion would be necessary to elucidate these possibilities. The expression of MIP-1α and MIP-2 on day 17 following intranasal ANDV challenge was similar across all treatment groups and substantially lower than that detected in ANDV-alone hamsters on day 10. Whether this reflects decreased numbers of AMθ at day 17 versus day 10 or whether this is a natural attempt by the hamster to downregulate lung inflammation has yet to be determined.

IL-8 (CXCL8) is another potent neutrophil chemoattractant that can be produced by a number of cell types in the respiratory tract, including activated alveolar macrophages, airway epithelial, airway smooth muscle, and airway endothelial cells responding to inflammatory stimuli (76, 77), but like MIP-1a, MIP-2, and MCP-1, there are only limited data on how these chemokines are regulated during human hantavirus infection. Neither Sin Nombre virus nor Hantaan virus induces IL-8, MCP-1, MIP-1a, or MIP-1b within 72 h of *in vitro* infection of human lung microvascular endothelial cells (78), and only HTNV induces IL-8 at times later than 72 h (79), suggesting that IL-8 may be more relevant to HFRS than to HPS. Analysis of sera from human HPS cases failed to detect changes in MIP-2, MIP-1a, IL-8, or MCP-1, although significant increases in granulocyte-macrophage colony-stimulating factor (GM-CSF) and M-CSF were detected (80). As myeloid cell differentiation, growth, and activation factors, GM-CSF and M-CSF could implicate such as macrophages and dendritic cells in disease (81, 82), although these results suggest that it is not necessarily due to the recruitment of other cell types by macrophages. Intriguingly, higher concentrations of IL-8 and GM-CSF in serum are found in female HFRS patients infected with PUUV than in their male counterparts, potentially linking IL-8 expression with HFRS severity (83). However, more males than females are likely to develop clinical HFRS following PUUV infection (84, 85) and there is no difference in HFRS disease severity in humans infected with PUUV (86), rendering the link between IL-8 and HFRS disease suspect. Whether chemokines such as MCP-1, MIP-1a, or MIP-2 follow a similar pattern of expression during human HFRS has yet to be determined.

We believe that this is the first report of hamster-specific ELISA kits from a commercial vendor to be used with the Syrian hamster animal model. Given the relatively short time interval between the publishing of the Syrian hamster genome and the commercial availability of these ELISA kits, we cannot discount the possibility that the cytokine expression patterns that we see following liposomal clodronate/control liposome treatment of ANDV-infected hamsters are related to the specificity of these kits. As such, while these measurements are reproducible, they should be interpreted within the context of the other parameters measured in these experiments (e.g., survival and AMθ numbers) until verified by other independent reports.

The potential role of VEGF in hantavirus disease pathogenesis has recently received a great deal of attention. *In vitro*, hantavirus infection sensitizes endothelial cells to VEGF, rendering them hyperpermeable (87) in a process involving VE-cadherin (88, 89) and potentially β3 integrin (90). Similarly, increased levels of VEGF can be detected in pleural effluent from patients with acute HPS (91) or in serum samples of acute-phase HFRS patients (92). Alveolar macrophages are known to express VEGF during pulmonary infection and other forms of acute lung injury (93–98), suggesting that AMθ may respond similarly to hantavirus infection. Still, the fact that all hamsters infected with ANDV developed disease (Fig. 2e and 3d) suggests that AMθ do not significantly influence the expression of VEGF in hamsters. A significant increase in VEGF expression in the BAL fluid of ANDV-infected hamsters late after infection (day 17) was observed compared to what was seen in normal hamsters, but as expected, the levels of VEGF detected in the BAL fluid of ANDV-infected animals were nearly identical at later times after infection (day 17) regardless of the presence or absence of AMθ (Fig. 6). However, contrary to our expectations, the depletion of AMθ resulted in a significant increase in the level of VEGF in the BAL fluid of ANDV-infected hamsters at early times after intranasal challenge. Interestingly, when macrophages were depleted in animals prior to intranasal challenge, those animals developed disease faster than either untreated ANDV-infected animals or animals receiving control liposome treatments. This would seem to support the suggestion that VEGF contributes to hantavirus disease, but it would argue that AMθ are not the sole source of VEGF. Pulmonary epithelial cells (99, 100) and neutrophils (101, 102) are other known sources of VEGF in the lung and could be contributing to the increased levels of VEGF seen in ANDV-infected hamsters. Epithelial cells are not the primary targets of hantaviruses and are thus unlikely to be expressing VEGF as a result of direct infection, but expression could be induced by inflammatory cytokines produced by other cells during infection or by hypoxia caused during HPS (103). Recently, a role for neutrophils in vascular leakage caused by HTNV infection of SCID mice has been suggested (104). Correspondingly, liposomal clodronate treatment resulted in a significant increase both in BAL fluid VEGF and in neutrophil numbers early after infection (day 10). Whether the depletion of neutrophils in hamsters alters hantavirus disease pathogenesis and the expression of VEGF in hamsters remains to be determined.

The role that immune cell types may play in disease pathogenesis may not be limited to direct antiviral responses or cytokines. As cells such as neutrophils, monocytes, and lymphocytes are recruited to sites of infection, they undergo the process of transendothelial cell migration, which is highly regulated by integrins, cadherins, and junctional adhesion molecules to prevent vascular leakage during the process (105, 106). Hantaviruses inactivate and dysregulate beta 3 integrins and VE-cadherin (88, 89), making it possible that infected endothelial cells may not be able to reform junctional complexes following paracellular diapedesis, leading to vascular leakage. Presumably, this would lead to visible gaps between endothelial cells, not borne out in electron microscopy (EM) analysis of hantavirus-infected endothelium from humans or hamsters (28, 33). Alternatively, neutrophils also possess the ability to migrate directly through endothelial cells via transcellular migration. During this process, endothelial cells form a dome, controlled by RhoA and F-actin (107), over the neutrophil to prevent vascular leakage. Endothelial cells to which polymorphonuclear leukocytes (PMNs) adhered often display many vesicles that can extend continuously between the cell membranes of the endothelial cells (108) similar to those seen in endothelial cells from hantavirus-infected hamsters (33). Dysregulation of dome formation could potentially allow for the direct flow of serum proteins through infected endothelial cells in a manner irrespective of noticeable gap formation in the endothelium.

Small-animal models are invaluable tools to study the pathogenesis of diseases caused by neglected infectious disease agents such as Andes virus. However, the utility of the hamster model, as well as the role of the immune response in hantavirus disease pathogenesis, is contentious. Previously, we and others have demonstrated that the ablation of adaptive T and B cell responses to ANDV infection in hamsters does not alter the course of disease (109, 110). Here, using the Syrian hamster/Andes virus lethal disease model, we demonstrate that another component of the immune system is not directly responsible for the HPS-like disease cause by ANDV in hamsters. The mechanism by which hantaviruses cause disease in humans and hamsters alike is not clear, and many mechanisms of disease, both immune related and virus intrinsic, have been proposed. Making this more difficult is that aspects of the immune response to hantavirus infection are likely to be important for protection and viral clearance, even as they are viewed as contributing to disease. Still, it will be necessary to continue to evaluate other immune cell types, as we seek to understand their role in contributing to disease or protection following hantavirus infection.
